# Characterization of structural stability of palm oil esters-based nanocosmeceuticals loaded with tocotrienol

**DOI:** 10.1186/1477-3155-11-27

**Published:** 2013-08-01

**Authors:** Sook Han Ng, Pei Meng Woi, Mahiran Basri, Zahariah Ismail

**Affiliations:** 1 School of Pharmacy, International Medical University, 57000, IMU Bukit Jalil, Kuala Lumpur, Malaysia; 2 Department of Chemistry, Faculty of Science, University of Malaya, 50603, Kuala Lumpur, Malaysia; 3 Department of Chemistry, Faculty of Science, Universiti Putra Malaysia, 43400, UPM Serdang, Selangor, Malaysia; 4 Institute of Bioscience, Universiti Putra Malaysia, 43400, UPM Serdang, Selangor, Malaysia; 5 Sime Darby Research Sdn. Bhd Carey Island, 42960, Pulau Carey, Selangor, Malaysia

**Keywords:** Palm oil esters, Tocotrienol, Nanoemulsion, Sedimentation, Rheological

## Abstract

**Background:**

Palm oil esters (POEs) are esters derived from palm oil and oleyl alcohol have great potential in the cosmetic and pharmaceutical industries due to the excellent wetting behavior of the esters without the oily feel. The role of oil-in-water nanoemulsions loaded with tocotrienol sedimentation behavior was studied. LUMiFuge® 116 particle separation analyzer was used to investigate the sedimentation behavior of POEs/tocotrienol/xanthan gum nanoemulsion system during centrifugation. Analyzing the sedimentation kinetics of dispersions in a centrifugal field also yields information about the rheological behavior and structural stability.

**Methods:**

Experiments were performed in an analytical centrifuge at 11×g to 1140×g (LUMiFuge® 116 particle separation analyzer). The samples in the LUMiFuge® 116 particle separation analyzer were centrifuged at 3000 rpm for 15 h at 32°C. Sample volume of 2 cm^3^ was used. The rheological property of nanoemulsions was investigated using oscillatory measurements test. A rotational/oscillatory viscometer, Kinexus Rheometer (Malvern Instrument, UK) was used. All measurements were performed with a stainless steel cone-plate sensor at 25.0 ± 0.1°C with 4°/40 mm.

**Results:**

The stable nanoemulsions showed sedimentation rates at earth gravity of 5.2, 3.0 and 2.6 mm/month for 10%, 20% and 30% (w/w) oil phase, respectively. Rheological behavior is an important target during the design of palm oil esters-based nanocosmeceuticals. The presence of a network structure was indicated by measurements which showed G’ to be greater than G”. This result implied the predominant elastic response and high storage stability of the nanoemulsion. It was also observed that the increase in oil phase concentration led to the profile which strongly indicated that the solid like elastic property; where the values of phase angle, δ of these nanoemulsions was lower than 45°.

**Conclusions:**

The nanoemulsions with higher oil phase concentration (30% (w/w)) showed greater elasticity which implied strong dynamic rigidity of the nanoemulsion. It was the most stable with longest shelf-life.

## Background

Cosmeceutical is a logical evolutionary concept, given the advances in skin anatomy and physiology. Contemporary belief is that almost all compounds applied to skin have the ability to penetrate and exert changes to skin structure. Currently, cosmeceutical-based products are very popular, with sales representing one of the largest growing segments of the skin care market, especially for products that are designed to help in the prevention and the treatment of aging skin [1]. Cosmeceuticals represent a new category of multifunctional products that rely on science and technology to deliver clinically proven active ingredients to the skin.

Palm oil is one of the most widely used plant oils in the world. It is produced from the fruit of oil palm (*Elaeis guineensis*), which is grown in mass plantation in tropical countries such as Malaysia, Indonesia and Nigeria. The oil consists of 95% triglycerides and 5% diglycerides whereby carbons of the carboxyls range from 10–20 with or without double bonds. Palm oil is mainly used for food applications, but it is also used in some other industries such as soap, detergent and cosmetic production [2]. Palm oil esters, derivative of palm oil, have excellent wetting behavior without the oily greasy feeling. Thus, palm oil esters are excellent ingredient to be used in cosmeceutical and pharmaceutical formulations. The potential of palm oil esters to be used in the formation of emulsions for delivery system in cosmeceutical and pharmaceutical formulations has never been reported before.

Nanoemulsions are emulsions with droplet size in the range 20–200 nm. They are independent of molecular size of the hydrophilic solute and the nature of the aqueous phase. In addition, nanoemulsions delivery system was independent of animal skin characteristics such as the stratum corneum thickness and the follicle-type [3]. Thus, nanoemulsions due to their extremely small size are suitable to be used as delivery system in cosmeceuticals. However, nanoemulsions are only kinetically stable and therefore, it is also a very fragile systems by nature [4]. As they are transparent and usually very fluid, the slightest sign of destabilization easily appears. They become opaque and creaming may be visible. Thus, stability of the nanoemulsion is a critical factor to be analysed. The achievement of developing long time stability of cosmetic products (3 years shelf life) is often difficult and costly deep in the development of new formulations.

Tocotrienol are fat-soluble vitamins related to the family of tocopherols. Vitamin E is a generic name describing compounds having bioactivities of both tocopherol and tocotrienol derivatives. Tocotrienols, are capable of scavenging and quenching reactive oxygen species, also known as free radicals. Their antioxidative activity, however resides mainly with its “chain-breaking” property which neutralizes peroxyl and alkoxyl radicals generated during lipid peroxidation [5].

This paper focuses on the sedimentation stability of dispersions using a recently introduced LUMiFuge® 116 particle separation analyzer. The system measures near infrared (NIR) transmission profiles continuously during centrifugation. Separation kinetics can thus be studied under accelerated conditions. Our primary aim was to apply the LUMiFuge® 116 particle separation analyzer method to mixtures of xanthan gum. Our secondary aim was to correlate the LUMiFuge® 116 particle separation analyzer data with parameters from static and dynamic rheology to elucidate their significance in terms of sedimentation stability.

## Materials and methods

### Raw materials and dispersion manufacture

Palm oil esters were obtained from our laboratory through enzymatic alcoholysis of palm oil with oleyl alcohol using Lipozyme RM IM as the catalyst [6]. Polyoxyethylene sorbitan monooleate (Tween 80) and sorbitan monooleate (Span 80) were purchased from Fluka Chemie GmbH, USA. Xanthan gum from Xanthomonas campestris was obtained from Fluka Chemie GmbH, France. Gold Tri. E 70 was from Golden Hope Bioganic, Malaysia.

### Methods

#### ***Selection of the nanoemulsion compositions***

Composition for nanoemulsion formulations with 5% surfactant and oil phase at 10%, 20% and 30% (w/w) were selected for fabrication of nanoemulsions system. The disperse phase comprises skin active ingredient such as tocotrienol. The continuous phase comprises components other than water including but not limited to thickener, anti-microbial and humectant to impart an increased benefit to the skin tissues.

#### ***Preparation of emulsions***

Emulsions were prepared using ultrasonic (UP400S Hielscher Sonifier, Germany) of 400 W nominal powers and a frequency of 24 kHz equipped with a 22 mm sonotrode tip. This was placed in a custom-built cooling jacket. Chilled water at 3°C was continuously passed through the jacket. Emulsions were prepared where both oil and aqueous phases were separately warmed up to 70 ± 5°C. Xanthan gum was dispersed in deionized water at 0.8% (w/w). An emulsion sample (100 mL) was prepared and homogenized at 6000 rpm for 5 min with a Polytron homogenizer (Kinematica GmbH, Germany) rotor stator. The temperature was lowered to 40°C. At 40°C, the active ingredients and preservative were added. The emulsions were further homogenized using ultrasonic cavitation for 5 min. The sonifier tip horn was adjusted to 2 cm below the surface of a 100 mL sample. Sonication was performed at acoustic amplitude of 20% and 0.5 cycles.

### Sedimentation behavior

The stability of the emulsion was examined with an optical centrifuge, LUMiFuge® 116 particle separation analyzer (L.U.M. GmbH, Germany). LUMiFuge® 116 particle separation analyzer technologies, based on optical centrifuge detect includes near IR optics. The instrument simulates comprehensive emulsion processes due to gravitation forces [7]. The instrument presents a typical graph for each process: creaming, sedimentation and phase separation. The data are integrated by the computer and the integration graph shows the percentage of light absorbance per hour – the “creaming rate”.

The rate is correlated to the stability of the emulsions whereby higher creaming rate indicates lower stability. The samples in the LUMiFuge® 116 particle separation analyzer were centrifuged at 3000 rpm for 15 h at 32°C. Sample volume of 2 cm^3^ was used. A light source (NIRLED) is radiated to the cells during centrifugation and its transmission through the sample was measured. The obtained transmission profiles measured between the top and bottom of the measurement cell enabled quantification of the dispersion quality. Quantitative data were available by integration of the transmission profiles. The profile was used to calculate the sedimentation or flotation velocity. In general, as more stable the dispersion, means the dispersibility, the smaller the sedimentation and clarification rate.

### Rheology

A rotational/ oscillatory viscometer, Kinexus Rheometer (Malvern Instrument, UK) was used. All measurements were performed with a stainless steel cone-plate sensor at 25.0 ± 0.1°C with 4°/40 mm.

Strain sweep measurements at 1 Hz were made with all samples to determine strain amplitude within the linear viscoelastic range. Subsequent oscillatory measurements were performed at a frequency range of 0.001 to 100 Hz. Storage modulus *G*’ (Pa), loss modulus G” (Pa), loss tangent and tan δ were evaluated.

## Results and discussion

### Sedimentation behavior

In order to obtain a more quantitative measure for the dispersion quality, we utilized a new method, which is based on measuring the sedimentation behavior of the nanoemulsions in the dispersion. Utilizing centrifugal forces enables acceleration of the sedimentation of the dispersed bundles to the bottom of the cell, leading to a clarification of the dispersion. The rate of clarification of the dispersion can therefore be used as a measure for dispersibility.

The sedimentation behavior under centrifugal force was evaluated using LUMIFuge® 116 particle separation analyzer despite no visual changes occurring upon the emulsions standing (at 1 g) over a period of several months. Particle migration due to centrifugal force results in a variation of the local particle concentration and correspondingly local and temporal variations of transmission occur. The evolution (kinetics) of the demixing was documented by a sequence of the respective transmission profiles (transmission versus position within the sample) taken after selected time intervals.

**Figure 1:**
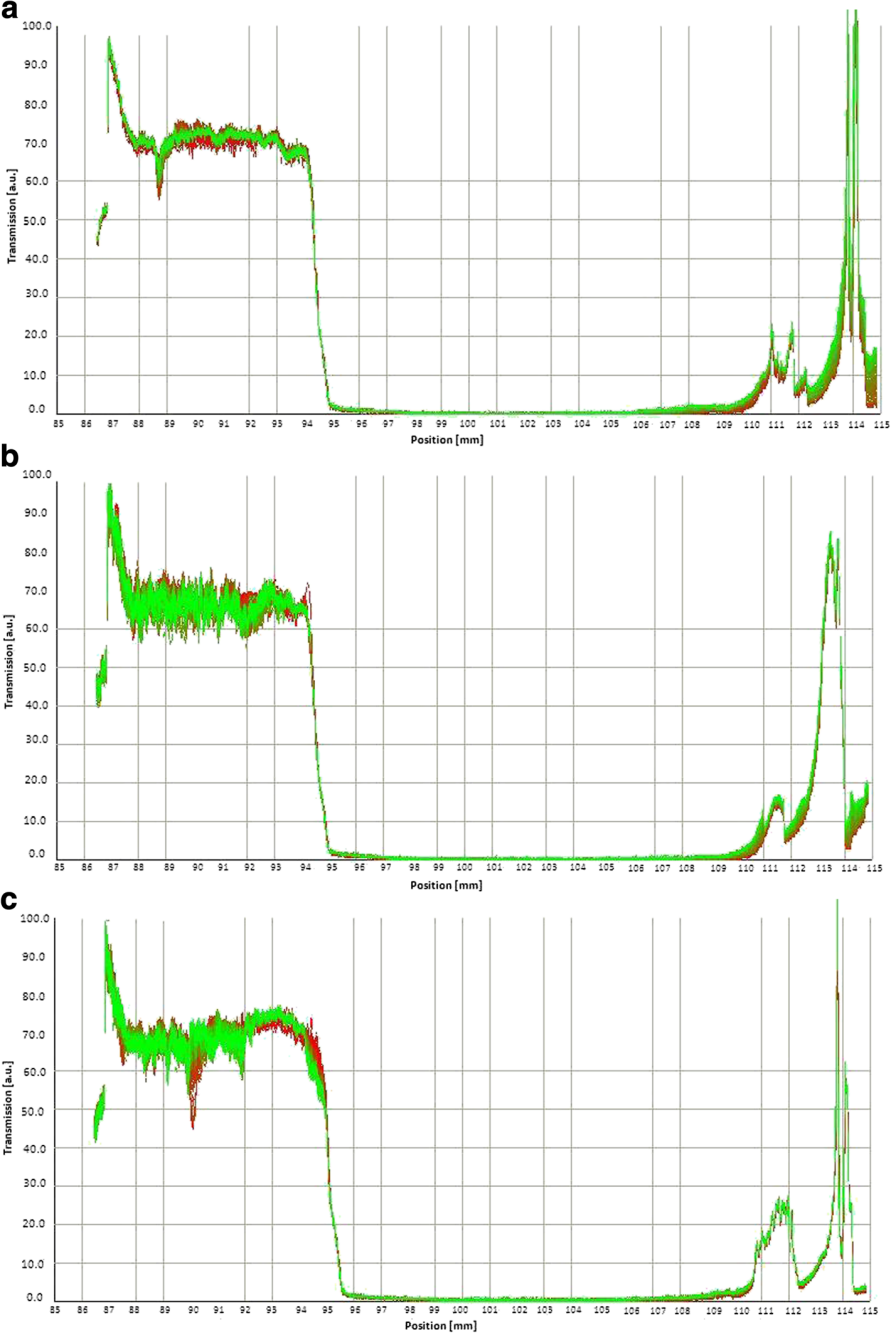
**Evolution of transmission profiles. (a)** Transmission profiles at 3000 rpm of 10% oil phase concentration, from the top of the cell (87 mm) to the bottom (115 mm) of the cell. **(b)** Transmission profiles at 3000 rpm of 20% oil phase concentration, from the top of the cell (87 mm) to the bottom (115 mm) of the cell. **(c)** Transmission profiles at 3000 rpm of 30% oil phase concentration, from the top of the cell (87 mm) to the bottom (115 mm) of the cell.

In a typical measurement, the light transmission was measured as a function of the position on the centrifuge tube. Performing this measurement while subjecting the sample to centrifugal forces yield a plot of percent transmission as a function of measurement time, giving an indication of the sedimentation rate in each sample. Figure 1(a), 1(b) and 1(c) display measurements of a series of transmission profiles under centrifugation forces at 3000 rpm for nanoemulsions with 10%, 20% and 30% (w/w) oil phase concentration, respectively.

The first profile (on the left) depicted the position of the interface immediately after the start of the centrifugation (20 s) and the second profile (on the right) after 54 315 s of centrifugation. The overlay of profiles on the right (thickening of the line) documented that the sedimentation process came to its end and marks the position of the sediment. During centrifugation, an increase in transmission was observed in all the positions, especially at the top of the sample cell (87–90 mm). The transmission at the top of the sample (87–90 mm) increased during centrifugation, but remained close to 0% in the rest of the cell.

The particles present at the position 90 mm and above remained dispersed in the medium even under centrifugal forces, and therefore no light was transmitted. The obtained sedimentation velocity was virtually independent from the setting of the “Trigger”. In the above case, the “Trigger” values of 30% of the maximum transmission sedimentation velocities were 0.0020 μm/s, 0.0012 μm/s and 0.0010 μm/s for nanoemulsions with 10%, 20% and 30% (w/w) oil phase concentration, respectively. It should be noted that under the centrifugation condition, sedimentation rates of 0.0020, 0.0012 and 0.0010 μm/s corresponded to sedimentation rates of dispersed particles at earth gravity (1 × g) of about 5.2, 3.0 and 2.6 mm/month in dispersion for nanoemulsions with 10%, 20% and 30% (w/w) oil phase concentration, respectively.

Migration of particles or droplets was recorded during centrifugation using this stability analyzer. Data may be assessed in terms of stability behavior. The quick and reliable classification of the stability and prediction of shelf life of new products under development, or actually processed products for the market, which demix under gravity only over a period of months or even years, would essentially shorten the development cycles and adds new possibilities for quality control. The “structural stability” as measured by analytical centrifugation correlates well with the yield stress obtained by rheological techniques. The overall results proved the high emulsion stability of the corresponding O/W emulsions.

### Oscillatory measurements: frequency sweep profile

**Figure 2:**
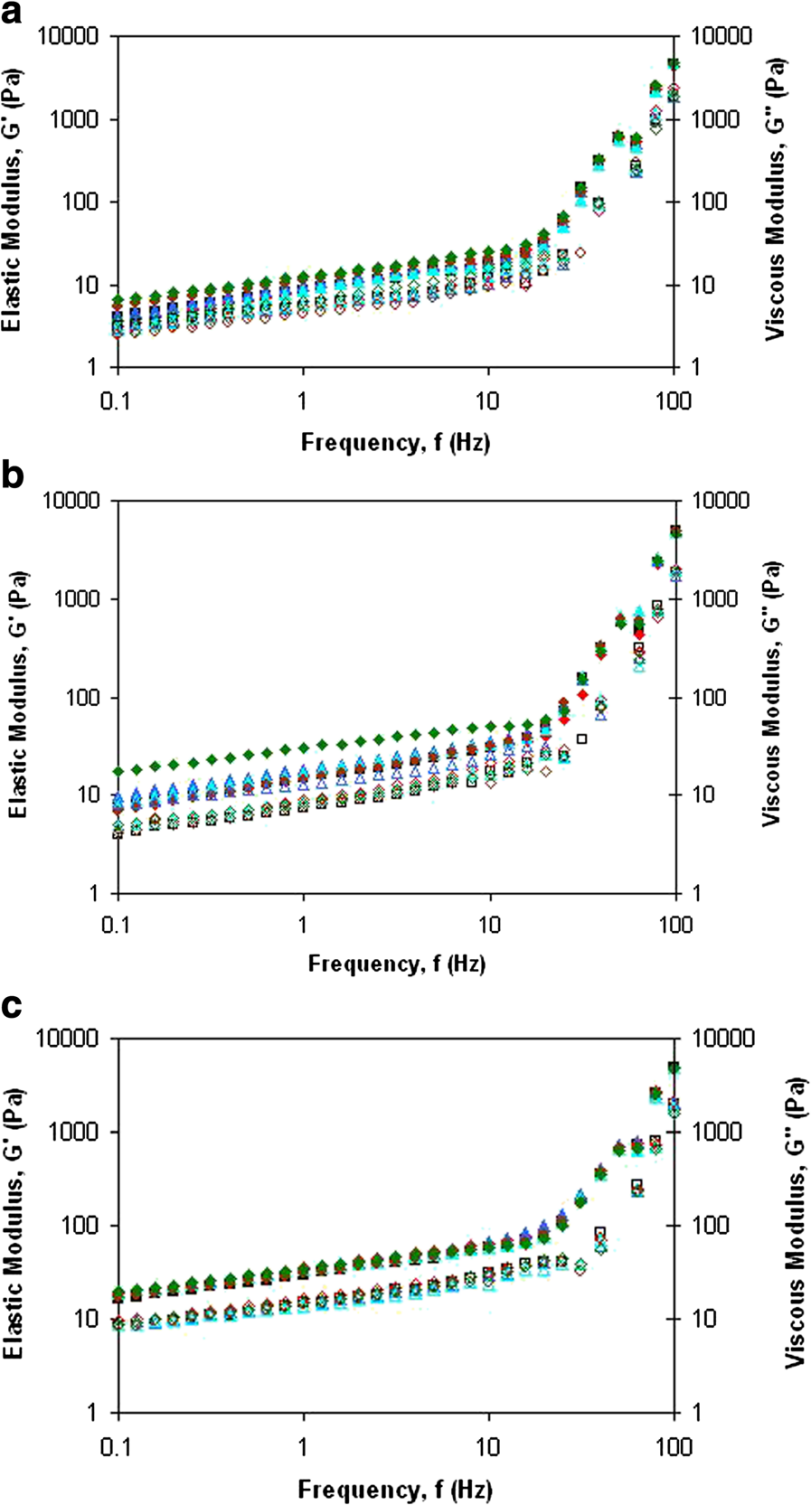
**Frequency sweep profile of emulsions. (a)** Frequency sweep profile of the emulsions for 10% oil phase concentration. The diagram shows the storage modulus, G’ (5% (black-shaded square), 6% (red diamond), 7% (blue-shaded triangle), 8% (sky-blue-shaded triangle), 9% (brown decagon) and 10% (green diamond)) and loss modulus, G” (5% (black square), 6% (red octagon), 7% (blue triangle), 8% (sky-blue triangle), 9% (brown circle) and 10% (green octagon)). **(b)** Frequency sweep profile of the emulsions for 20% oil phase concentration. The diagram shows the storage modulus, G’ (5% (black-shaded square), 6% (red diamond), 7% (blue-shaded triangle), 8% (sky-blue-shaded triangle), 9% (brown decagon) and 10% (green diamond)) and loss modulus, G” (5% (black square), 6% (red octagon), 7% (blue triangle), 8% (sky-blue triangle), 9% (brown circle) and 10% (green octagon)). **(c)** Frequency sweep profile of the emulsions for 30% oil phase concentration. The diagram shows the storage modulus, G’ (5% (black-shaded square), 6% (red diamond), 7% (blue-shaded triangle), 8% (sky-blue-shaded triangle), 9% (brown decagon) and 10% (green diamond)) and loss modulus, G” (5% (black square), 6% (red octagon), 7% (blue triangle), 8% (sky-blue triangle), 9% (brown circle) and 10% (green octagon)).

The oscillation rheology data are of special interest as these experiments were performed in the linear viscoelastic range. Figure 2(a), 2(b) and 2(c) display a frequency sweep from a comparatively stable sample. Profiles depicting frequency dependence of storage modulus, *G*’ and the loss modulus, *G*” as a function of oil concentration. These profiles suggested that, both *G*’ and *G*” responses increased with frequency and surfactant concentration. The presence of a network structure was indicated by measurements which showed *G*’ to be greater than *G*”. This behavior was readily demonstrated in Figure 2(a), 2(b) and 2(c). This result implied the predominant elastic response and high storage stability of the nanoemulsion. This also suggested that very little energy was dissipated by these nanoemulsions. It also meant that at the given surfactant concentration domain the solid like elastic property of these nanoemulsions was dominant over their liquid like viscous property. These frequency dynamic moduli relationships further suggested that not only these nanoemulsions spread easily but that their storage stability was reasonably high [8].

The slow increase of *G*’ with frequency indicated that flocculation was not significant in these nanoemulsions. However, the fact that *G*’ increased with the increase in both surfactant and oil concentration showed that the strength of inter particle interaction played important role in these systems, and that it increased with decreasing droplet size. No domination of the *G*” over the *G*’ was observed, indicating that the applied stress was able to be stored in the elastic component although at low stress condition. This proved that the presence of strong interactions among the nanoemulsion droplets. Tadros (1992) [9] described that *G*’ is a measure of the energy stored elastically in the system, while *G*” is the measure of the energy dissipated as heat during viscous flow. The input energy which comes from the shear stress is stored elastically and represented as *G*’. Therefore, a decrease in the magnitude of the viscous component (*G*”) is observed. When the frequency was further decreased, most of the input energy was no longer able to be stored, but will be dissipated through viscous flow [10].

**Figure 3:**
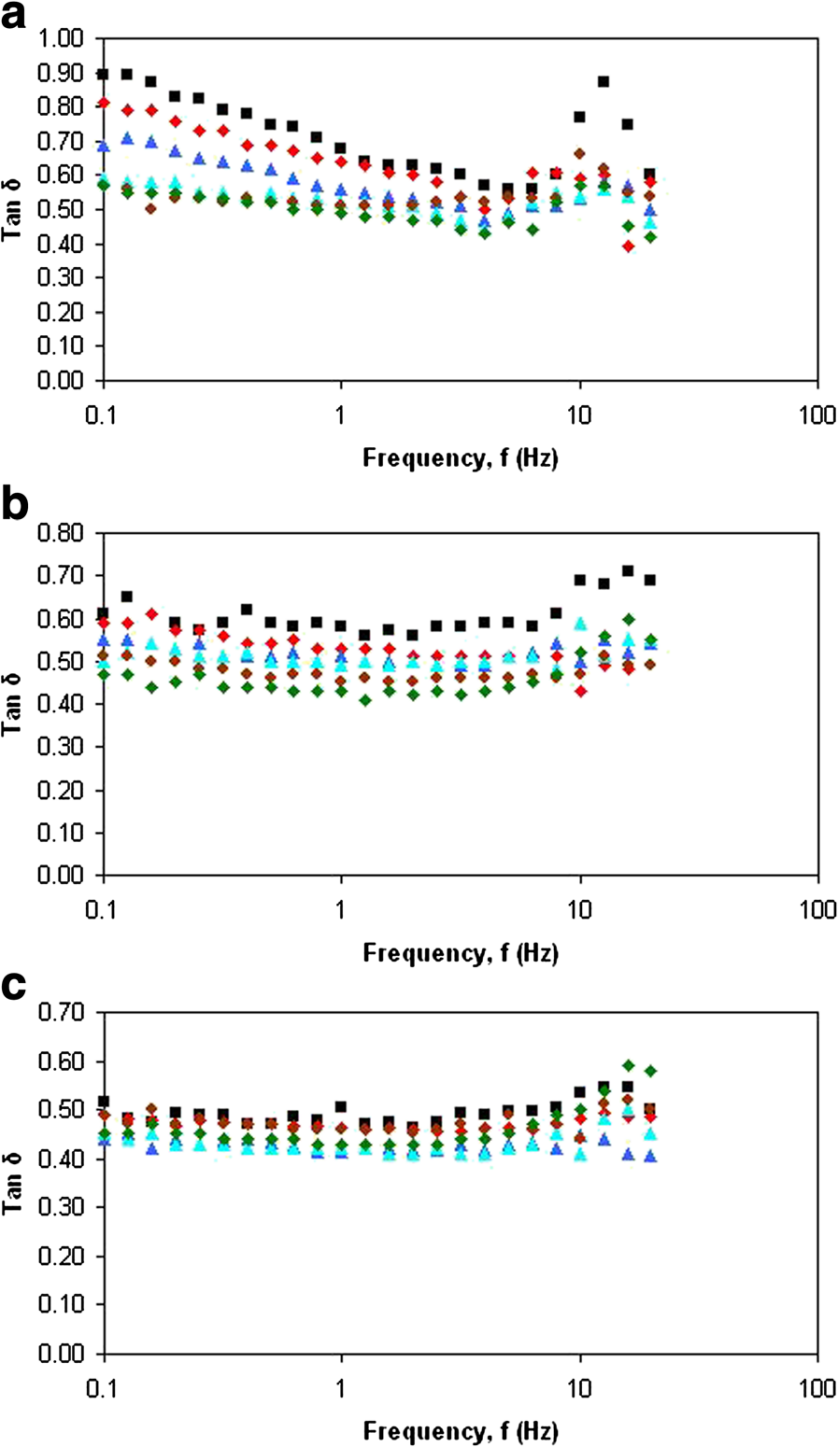
**The effect of Tan δ on frequency (Hz). (a)** The Tan δ of the emulsions on frequency (Hz) for 10% oil phase concentration. Surfactant concentration; 5% (black-shaded square), 6% (red diamond), 7% (blue-shaded triangle), 8% (sky-blue-shaded triangle), 9% (brown decagon) and 10% (green diamond). **(b)** The Tan δ of the emulsions on frequency (Hz) for 20% oil phase concentration. Surfactant concentration; 5% (black-shaded square), 6% (red diamond), 7% (blue-shaded triangle), 8% (sky-blue-shaded triangle), 9% (brown decagon) and 10% (green diamond). **(c)** The Tan δ of the emulsions on frequency (Hz) for 30% oil phase concentration. Surfactant concentration; 5% (black-shaded square), 6% (red diamond), 7% (blue-shaded triangle), 8% (sky-blue-shaded triangle), 9% (brown decagon) and 10% (green diamond).

Practically, it was not easy to judge whether the solid like elastic behavior of these nanoemulsions dominated over their liquid like viscous behavior from these frequency dependence of dynamic moduli alone. A better picture as to how these emulsions behave under a small amplitude oscillatory shear can be obtained by examining frequency dependence of Tan δ as shown in Figure 3(a), 3(b) and 3(c). Tan δ measures the relative magnitude of the *G*’ and *G*” [11]. Emulsions with Tan δ higher than 1 is characterized as liquid like, while a solid like emulsion will have Tan δ less than 1, and the perfect viscoelastic material will have Tan δ equal to 1.

Irrespective of both surfactant oil concentration, the fact that the values of phase angle, δ of these nanoemulsions was lower than 45°, it was evident that the solid like elastic property dominated in these nanoemulsions over their liquid like viscous property. Values for δ provide information about the nature of the viscoelastic responsed of the nanoemulsion system. In elastic networks δ is 0°, whereas in purely viscous liquids δ is 90°. For viscoelastic systems δ takes some value in this range. The closer δ is to 0° the more the nanoemulsion system displays an elastic response to the application of the shear stress and thus the more developed was the gel-like colloidal network.

**Figure 4:**
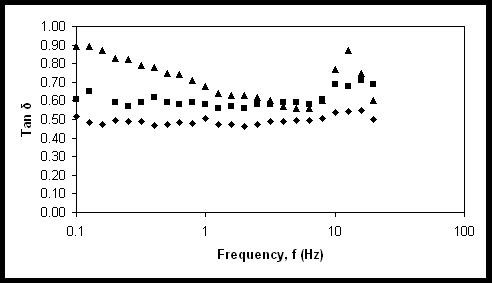
**The effect of Tan δ on frequency (Hz).** Emulsions with 10% (black-shaded triangle), 20% (black-shaded square), 30% (black octagon) oil phase concentration.

Figure 4 shows the effect of oil phase concentration to the Tan δ of nanoemulsions. It was observed that the increase in oil phase concentration led to the profile which strongly indicated that the solid like elastic property. The increase of elasticity when oil phase concentration was increased could be explained on the effect of droplet concentration. According to Tadros (1996) [11], the elastic modulus (*G*’) attributed from the hydrodynamic and surface force was very dependent to the surface-to-surface separation distance. These interactions between droplets become stronger when the droplets are getting closer to each other.

At lower oil concentration, the droplets separation distances were large and comparable to droplet radius. The droplets were loosely packed and were still able to diffuse with slower rate. Due to large separation distances between the droplets, the interaction among the droplets was relatively weak. The increase in oil phase concentration resulted in the increase of droplet concentration and decrease in the interdroplet separation distance. As the interdroplet separation distance decreased, some overlap of the interfacial layer occured. That caused rapid increase of the repulsive forces and eventually the droplets repelled each other.

When the droplets were in close distance, the hydrodynamic interaction acted as a repulsive force thus preventing the droplets from coming into close contact. Meanwhile, the attractive forces were increased by pulling the droplets towards each other. These interdroplet forces therefore acted like a spring holding the droplets and the droplets vibrated with small amplitude which showed the elastic property [12].

Tadros (1996) [11] stated that in such concentrated condition, the droplets interacted with many other droplets and the repulsive force produced a specific order among the droplets to the extent that a highly develop structure was reached. However, the droplets would merge or ruptured at high shear stress region when the droplets were too close to each other whereby the attractive forces were greater than the repulsive forces. The loss of elasticity can be observed by showing a decrease in magnitude of *G*’. Fortunately, the viscoelastic profiles of the nanoemulsions did not show this phenomenon, which indicated the structural rigidity and high droplet stability although subjected to high stress.

## Conclusions

Rapid assessment of physical stability is extremely valuable in preselecting formulations for conventional pharmaceutical stability testing. More focused long-term stability testing creates considerable savings while minimizing the risk of separation problems emerging at a late phase of formulation development. Rheological methods have the potential to screen physically stable systems. Also, rheological parameters remain only an indirect measure of physical stability; what is important is correlation with a real separation process. The results of this study indicate that rotational rheometry provides the requisite characterization of flow and appears suited to mirroring separation processes in a centrifugal field. In this study, the dynamic rheological method provided an alternative to the LUMIFuge® 116 particle separation analyzer method.

## Abbreviations

G’: Storage modulus; G”: Loss modulus; O/W: Oil-in-water; POEs: Palm oil esters; rpm: Revolutions per minute; s: Second; μm: Micrometre; w/w: Weight per weight.

## Competing interests

The authors declare that they have no competing interests.

## Authors’ contributions

NSH conceived of the study in the design of structural stability of palm oil esters-based nanoemulsions loaded with tocotrienol and drafted the manuscript. WPM carried out the investigation using oscillatory measurements test on rheological property of nanoemulsions. MB participated in the design and coordination and helped to draft the manuscript. ZI performed sedimentation behavior study. All authors read and approved the final manuscript.
